# Supplementary material for: Trans-Endothelial Migration of Memory T Cells Is Impaired in Alemtuzumab-Treated Multiple Sclerosis Patients
Source: J Clin Med. 2022 Oct 24;11(21):6266. doi: 10.3390/jcm11216266 (PMC9658536; doi:10.3390/jcm11216266)
Supplement: Supplementary file 1 [file jcm-11-06266-s001.zip › jcm-1943949 - supplementary.pdf]

Journal of Clinical Medicine

MDPI

## Supplementary Materials

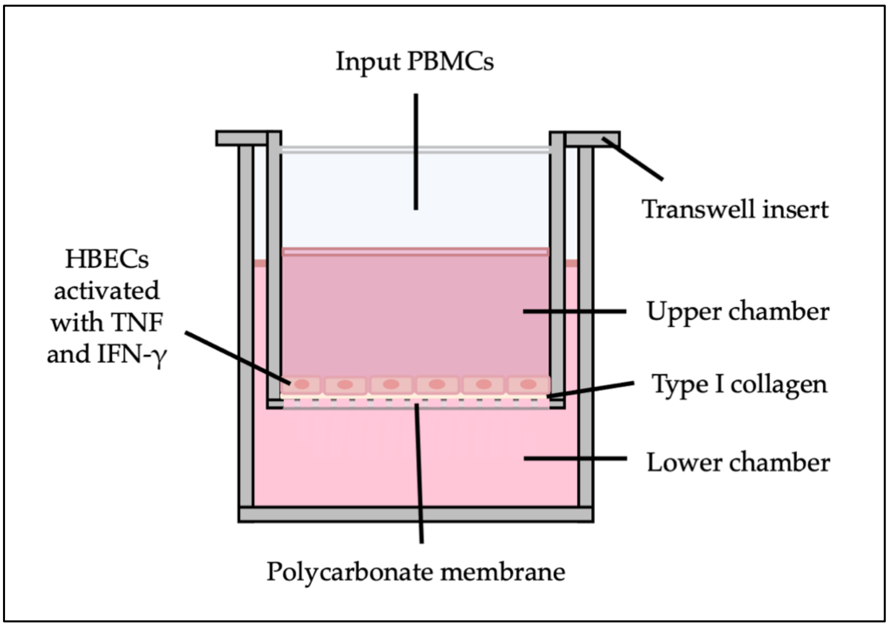


**Figure S1. Experimental set-up of in vitro BBB model using a transmigration assay.** The two-compartment system was separated by a porous membrane to replicate the peripheral blood circulation (upper chamber) and the CNS parenchyma (lower chamber). The polycarbonate membrane had 3 µm pores that were coated in collagen to mimic the basement membrane within the BBB. Collagen allowed HBECs, derived from the hCMEC/D3 cell line, to grow as a continuous monolayer. 5 ng/mL TNF and 10 ng/mL IFN-γ stimulated the endothelial cell layer to mimic an inflamed BBB. After cytokines were washed away, PBMCs that were isolated from healthy controls, untreated RRMS patients or alemtuzumab-treated RRMS patients were then added to the upper chamber and left overnight to migrate. HBEC, human brain endothelial cells; IFN-γ, interferon-gamma; PBMC, peripheral blood mononuclear cells; TNF, tumour necrosis factor.

**Table S1.** The inclusion and exclusion criteria of the study participants.

| Type of Study Participant | Inclusion Criteria | Exclusion Criteria |
|---|---|---|
| Healthy controls | • MS patients refer a friend or relative of a similar age<br>• People from the general population can make a voluntary contribution | • Methylprednisolone within the last 6 months<br>• Pregnancy |
| Untreated RRMS patients | • Either recruited from the MS Clinic at Sydney Neurology at the Brain and Mind Centre (BMC), Sydney, and from Central West Neurology and Neurosurgery in Orange, Central Western NSW<br>• At least yearly MRI on high resolution 3T magnets<br>• Have serial volumetric assessments performed at the Sydney Neuroimaging Analysis Centre (SNAC)<br>• Early non-treated patients with longstanding (>10 years) or benign RRMS | • Methylprednisolone within the last 6 months<br>• Pregnancy |

| | | |
|---|---|---|
| Alemtuzumab-treated patients | • Either recruited from the MS Clinic at Sydney Neurology at the Brain and Mind Centre (BMC), Sydney, and from Central West Neurology and Neurosurgery in Orange, Central Western NSW<br>• At least yearly MRI on high resolution 3T magnets<br>• Have serial volumetric assessments performed at the Sydney Neuroimaging Analysis Centre (SNAC)<br>• Received first dose of alemtuzumab treatment with additional follow-ups at 6 and 12 months | • Methylprednisolone within the last 6 months<br>• Pregnancy |

**Table S2.** List of fluorochrome-conjugated antibodies and clones for flow cytometric analysis.

| Cell Marker | Fluorochrome | Clone | Company |
|---|---|---|---|
| CD3 | Alexa Fluor 532 | UCHT1 | eBioscience, ThermoFisher Scientific, Waltham, MA, USA |
| CD4 | Alexa Fluor 700 | RPA-T4 | BioLegend, San Diego, CA, USA |
| CD8 | BV480 | RPA-T8 | BD Biosciences, Franklin Lakes, NJ, USA |
| CD14 | Pacific Blue | M5E2 | BioLegend |
| CD16 | FITC | 3G8 | Beckman Coulter, Lane Cove West, N.S.W., Australia |
| CD19 | BV570 | HIB19 | BioLegend |
| CD20 | Super Bright 436 | 2H7 | eBioscience, ThermoFisher Scientific |
| CD24 | PerCP | ML5 | BioLegend |
| CD27 | BV650 | O323 | BioLegend |
| CD28 | BV510 | CD28.2 | BioLegend |
| CD38 | BV785 | HIT2 | BioLegend |
| CD45RA | PerCP-Cy5 | HI100 | BioLegend |
| CD49d | PE-Cy5.5 | 9F10 | BioLegend |
| CD56 | BV750 | 5.1H11 | BioLegend |
| CD62L | BV711 | DREG-56 | BioLegend |
| CD69 | BV421 | FN50 | BioLegend |
| CD161 | PE-Dazzle 594 | HP-3G10 | BioLegend |
| CD196 | BV605 | GO34E3 | BioLegend |
| CD197 | PE | G043H7 | Beckman Coulter |
| CD274 | PECy7 | 29E.2A3 | BioLegend |
| GPR56 | APC | 4C3 | BioLegend |

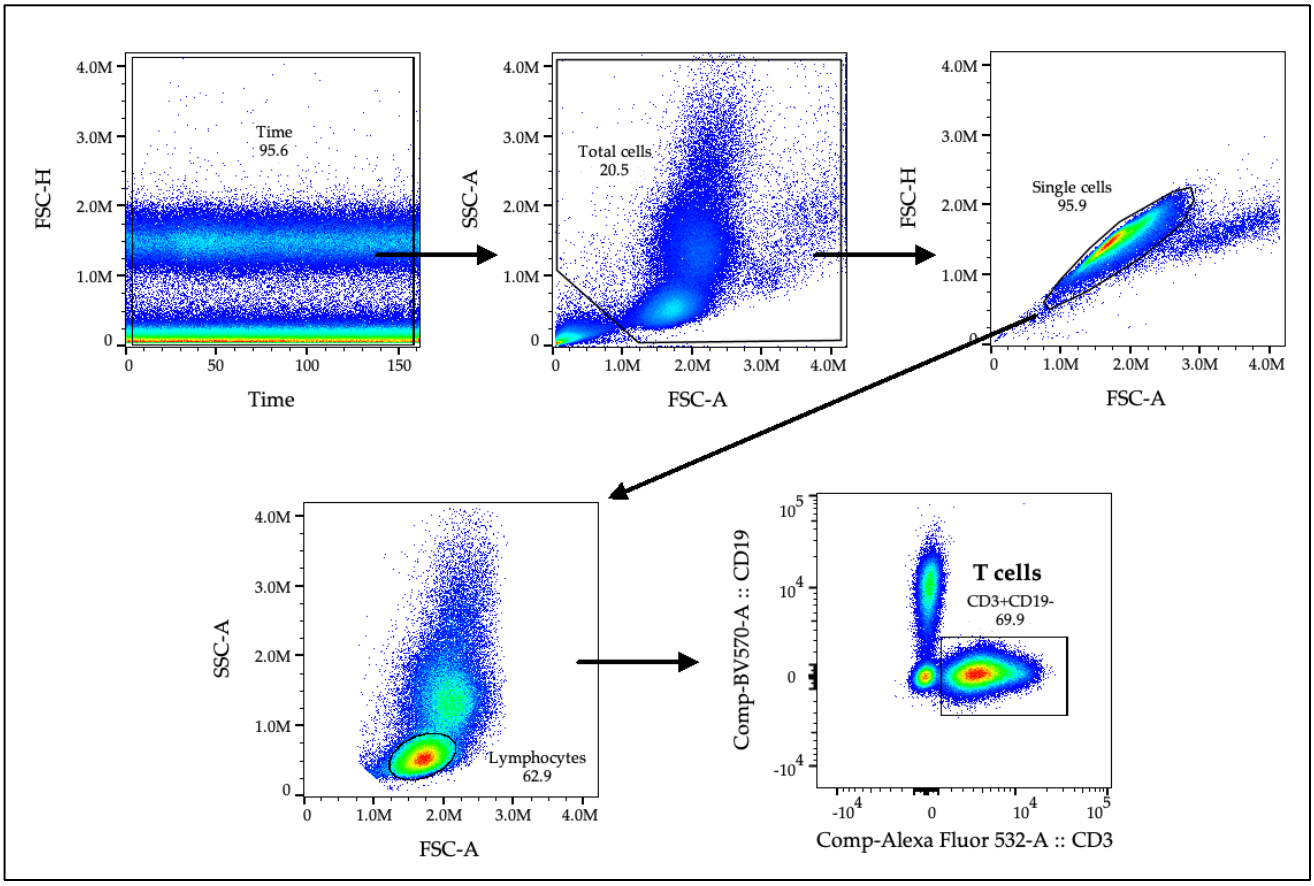


**Figure S2. Preliminary gating strategy.** Samples were first gated based on their forward-scatter-height (FSC-H) and time to obtain a consistent representative sample. Total PBMCs were gated based on their forward-scatter-area (FSC-A) and side-scatter-area (SSC-A). Single cells were identified by the exclusion of doublets and triplets using FSC-A and FSC-H. Lymphocytes were gated on using FSC-A and SSC-A. Major lymphocyte populations were identified and $CD3^+$ T cells were categorised based on $CD4^+$ and $CD8^+$ expression. PBMC, peripheral blood mononuclear cells.

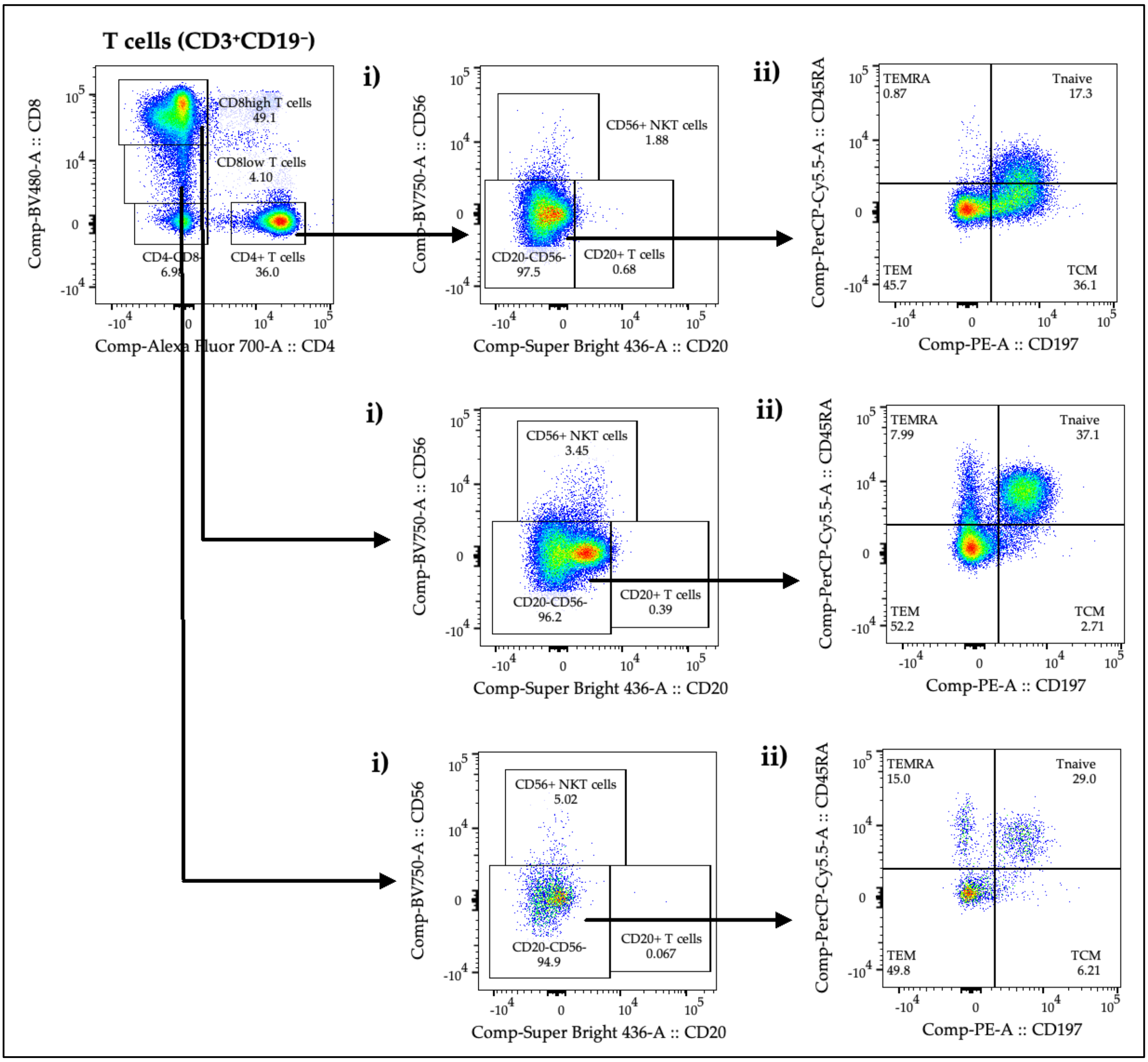


**Figure S3. Gating strategy of T cell subsets.** T cells were identified as $CD3^{+}CD19^{-}$; then divided into $CD4^{+}$, $CD8^{high}$ and $CD8^{low}$ T cell populations. The sequence of gating events is indicated by arrows to identify **(i)** NKT ($CD20^{-}CD56^{+}$), $CD20^{+}$ T cells ($CD20^{+}CD56^{-}$) and **(ii)** $T^{EMRA}$ ($CD45RA^{+}CD197^{-}$), $T^{naïve}$ ($CD45RA^{+}CD197^{+}$), $T^{CM}$ ($CD45RA^{-}CD197^{+}$) and $T^{EM}$ ($CD45RA^{-}CD197^{-}$) cells. NKT, natural killer T cells; $T^{EM}$, effector memory T cells; $T^{EMRA}$, terminally differentiated effector memory cells re-expressing CD45RA T cells; $T^{naïve}$, naïve T cells; $T^{CM}$, central memory T cells.

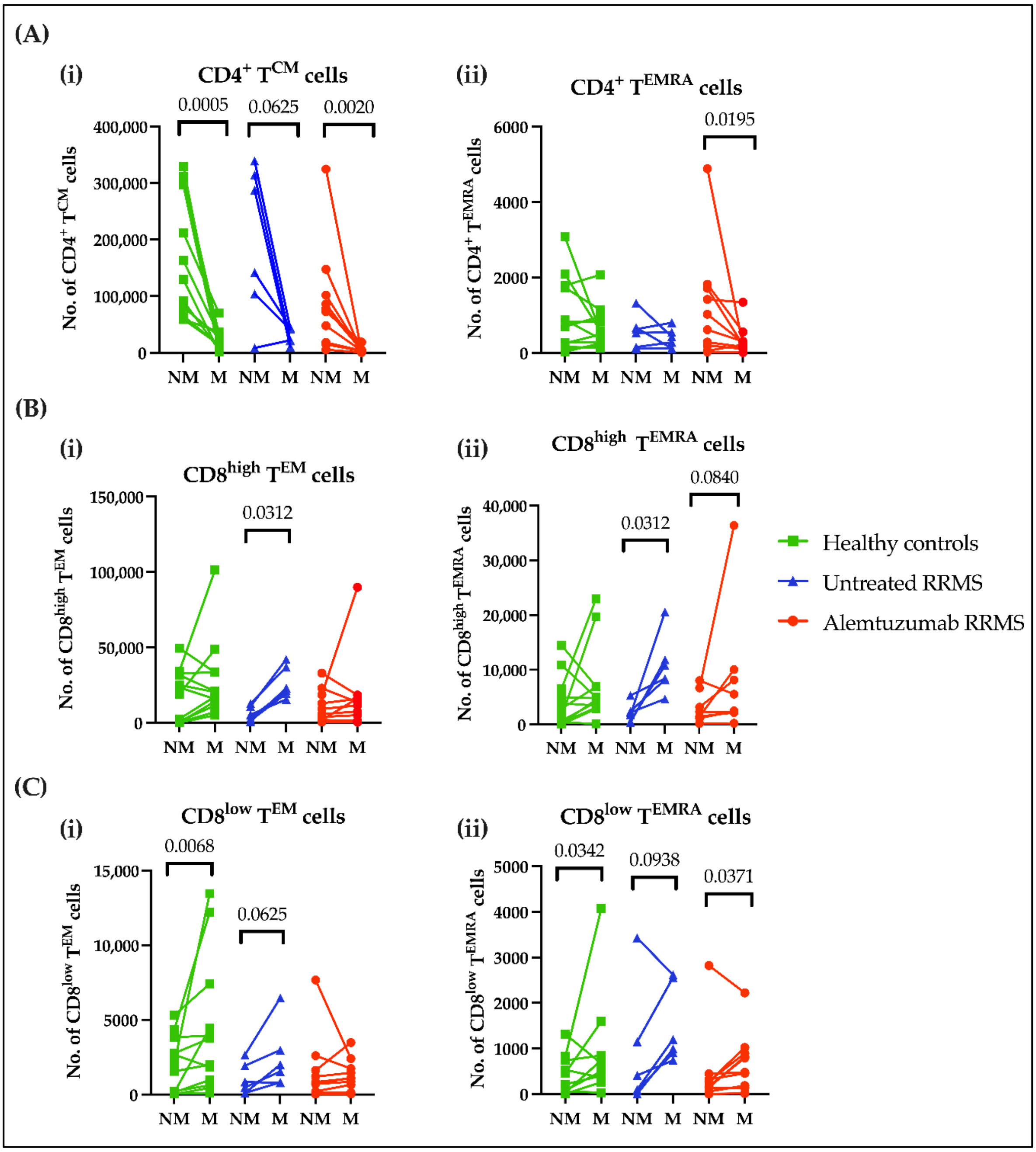


**Figure S4. The trans-endothelial migration of other CD4⁺ and CD8⁺ T cells subsets.** PBMCs were isolated from healthy controls (n = 12, green squares), untreated RRMS (n = 6, blue triangles) and alemtuzumab-treated RRMS patients (n = 10, red circles). After stimulation of the HBEC monolayer, PBMCs were added to the transwell assay and were left overnight to migrate. **(A)** The number of **(i)** CD4⁺ $T^{CM}$ cells and **(ii)** CD4⁺ $T^{EMRA}$. **(B)** The number of **(i)** CD8$^{high}$ $T^{EM}$ and **(ii)** CD8$^{high}$ $T^{EMRA}$ cells. **(C)** The number of **(i)** CD8$^{low}$ $T^{EM}$ cells and **(ii)** CD8$^{low}$ $T^{EMRA}$ cells. Wilcoxon matched-pairs signed-rank test. $p \leq 0.1$ are shown. NM, non-migrated cells; M, migrated cells; RRMS, relapsing-remitting multiple sclerosis; PBMC, peripheral blood mononuclear cell; HC, healthy control; HBEC, human brain endothelial cell. $T^{CM}$, central memory T cells (CD45RA$^-$CD197$^+$); $T^{EM}$, effector memory T cells (CD45RA$^-$CD197$^-$); $T^{EMRA}$, terminally differentiated effector memory T cells re-expressing expressing CD45RA (CD45RA$^+$CD197$^-$); $T^{naïve}$, naïve T cells (CD45RA$^+$CD197$^+$).

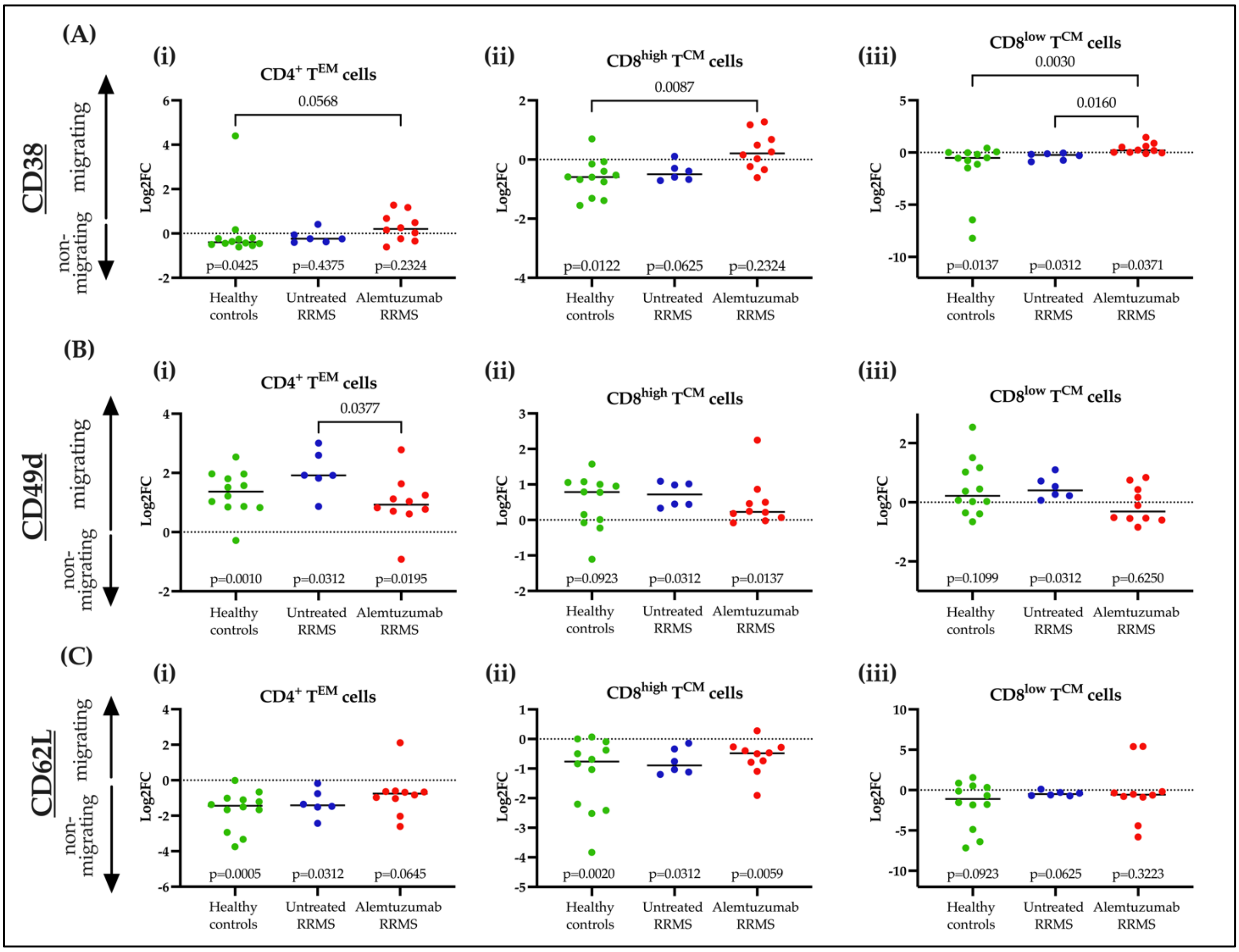


**Figure S5. The logarithm of 2-fold change (Log2FC) ratios of cell surface markers on $CD4^+$ and $CD8^+$ memory T cells.** The expression of cell surface markers was calculated in healthy controls (n = 12, green squares), untreated RRMS (n = 6, blue triangles) and alemtuzumab-treated RRMS patients (n = 10, red circles). **(A)** CD38 expression on **(i)** $CD4^+$ $T^{EM}$ cells, **(ii)** $CD8^{high}$ $T^{CM}$ cells and **(iii)** $CD8^{low}$ $T^{CM}$ cells. **(B)** CD49d expression on **(i)** $CD4^+$ $T^{EM}$ cells, **(ii)** $CD8^{high}$ $T^{CM}$ cells and **(iii)** $CD8^{low}$ $T^{CM}$ cells. **(C)** CD62L expression on **(i)** $CD4^+$ $T^{EM}$ cells, **(ii)** $CD8^{high}$ $T^{CM}$ cells and **(iii)** $CD8^{low}$ $T^{CM}$ cells. Kruskal-Wallis nonparametric one-way ANOVA with Dunn's multiple comparisons test was done between groups, with p-values shown above data. A one sample nonparametric Wilcoxon signed-rank test with Pratt method was done to compare individual values, with p-values shown below data. Wilcoxon signed-rank test with method of Pratt, where above 0 indicates higher expression in migrating cells and below 0 indicates higher expression in non-migrating cells. Median and $p \leq 0.1$ are shown. $T^{CM}$, central memory T cell; $T^{EM}$, effector memory T cell; RRMS, relapsing-remitting multiple sclerosis.

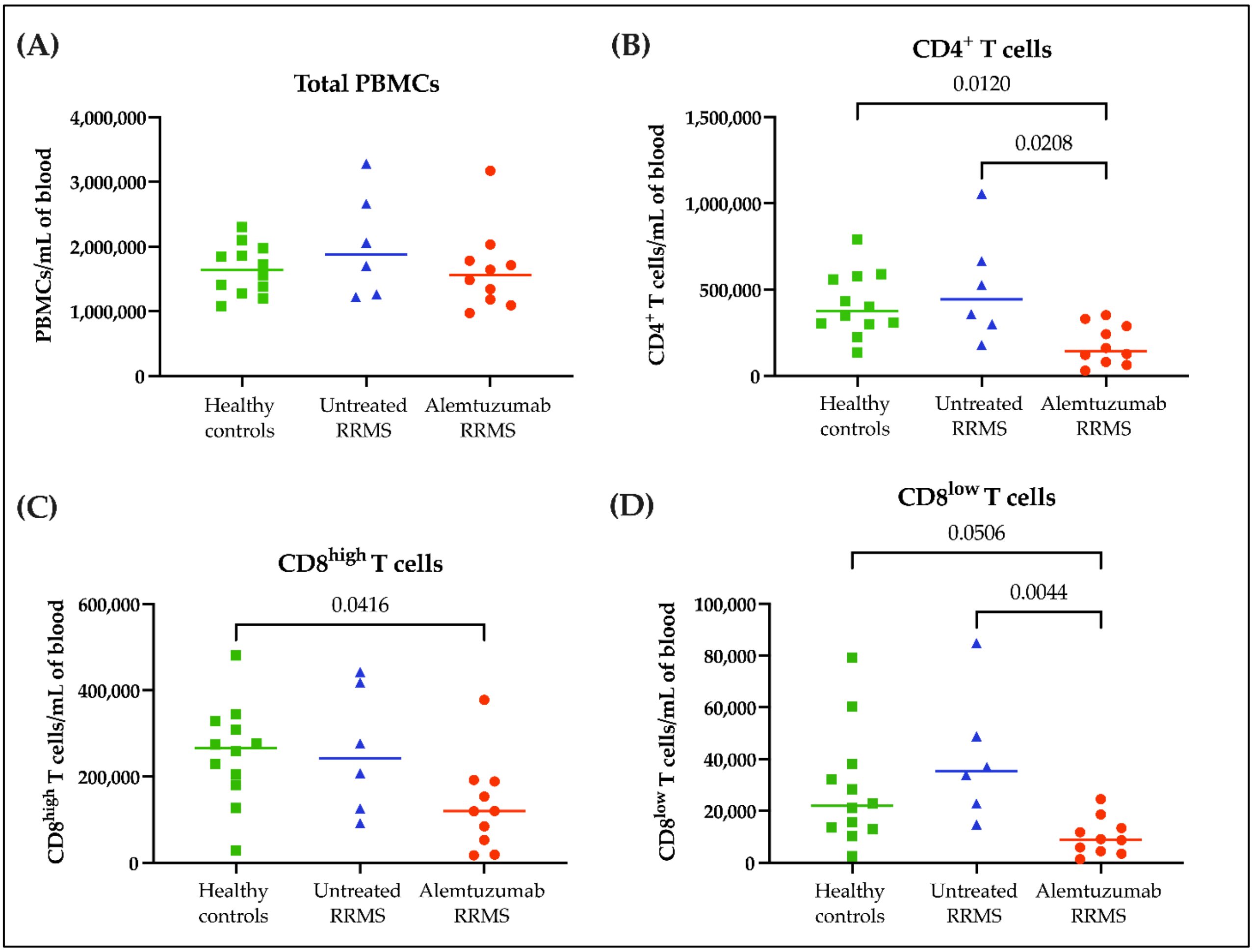


**Figure S6. Fresh cell counts of total PBMCs and major lymphocyte populations.** Blood was sampled from healthy controls (n = 12, green squares), untreated RRMS (n = 6, blue triangles) and alemtuzumab-treated RRMS patients (n = 10, red circles). Upon sample collection, fresh PBMCs were immediately isolated and phenotyped. **(A)** The total number of PBMCs per mL of blood. **(B)** The total number of $CD4^+$ T cells per mL of blood. **(C)** The total number of $CD8^{high}$ T cells per mL of blood. **(D)** The total number of $CD8^{low}$ T cells per mL of blood. Kruskal-Wallis nonparametric one-way ANOVA with Dunn's multiple comparisons test. Median and $p \leq 0.1$ are shown. PBMC, peripheral blood mononuclear cells; RRMS, relapsing-remitting multiple sclerosis.

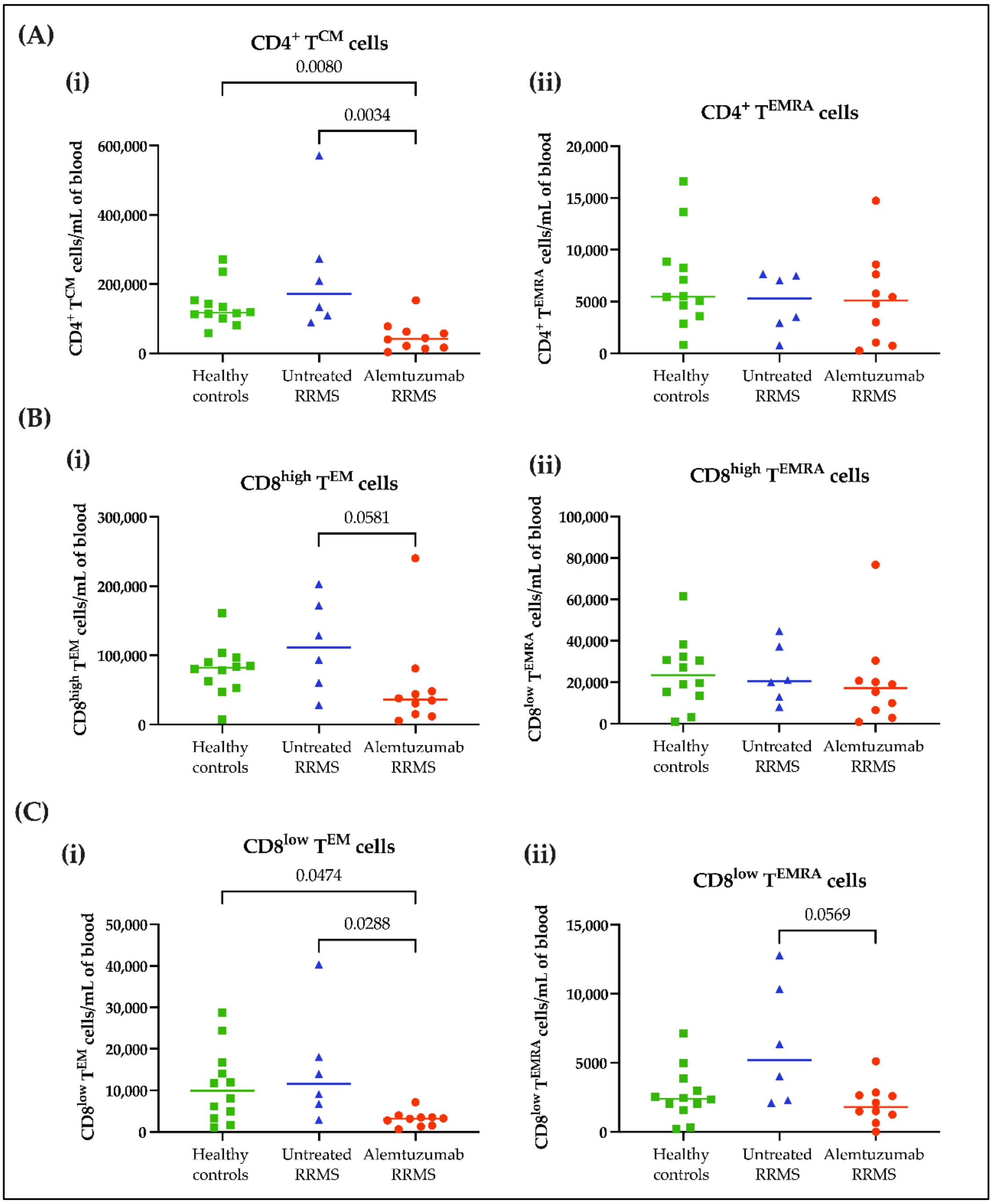


**Figure S7. Fresh cell counts of CD4+ and CD8+ T cell subsets.** Blood was sampled from healthy controls (n = 12, green squares), untreated RRMS (n = 6, blue triangles) and alemtuzumab-treated RRMS patients (n = 10, red circles). Upon sample collection, fresh PBMCs were immediately isolated and phenotyped. **(A)** The total number of **(i)** CD4+ T$^{CM}$ cells and **(ii)** CD4+ T$^{EMRA}$ cells per mL of blood. **(B)** The total number of **(i)** CD8$^{high}$ T$^{EM}$ cells and **(i)** CD8$^{high}$ T$^{EMRA}$ cells per mL of blood. **(C)** The total number of **(i)** CD8$^{low}$ T$^{EM}$ cells and **(i)** CD8$^{low}$ T$^{EMRA}$ cells per mL of blood. Kruskal-Wallis nonparametric one-way ANOVA with Dunn's multiple comparisons test. Median and p ≤ 0.1 are shown. PBMC, peripheral blood mononuclear cells; RRMS, relapsing-remitting multiple sclerosis; T$^{CM}$, central memory T cell; T$^{EM}$, effector memory T cell; T$^{EMRA}$, terminally differentiated effector memory T cells re-expressing expressing CD45RA (CD45RA+CD197-).
